# The Pygmalion Effect to Piano Teaching From the Perspective of Educational Psychology

**DOI:** 10.3389/fpsyg.2021.690677

**Published:** 2021-08-10

**Authors:** Wen Qi, Xingru Dong, Xiaoran Xue

**Affiliations:** ^1^College of Dance, Minzu University of China, Beijing, China; ^2^Academy of Music, Linyi University, Linyi, China; ^3^School of Sociology and Psychology, Central University of Finance and Economics, Beijing, China

**Keywords:** educational psychology, piano teaching, experimental research, Pygmalion effect, teachers' encouragement

## Abstract

Aiming at the problem of low student enthusiasm in piano teaching, this work tried to apply “Pygmalion effect” to piano teaching from the perspective of educational psychology. Forty-five students were chosen from nine classes in grades 2, 4, and 6 of a central elementary school in Beijing City using experimental research, and the experimental group and the control group were set up. A questionnaire was adopted to conduct the pre-test and post-test of the experiment, which were done to analyze the factors that affect the attitude of students toward music learning and the effect of piano teaching. The results show that the encouragement level of the piano teacher and the enthusiasm of the students in the piano course were significantly correlated at the 0.01 level. There was also a significant correlation between self-efficacy and student piano learning enthusiasm at the 0.01 level. Furthermore, the pre-test and post-test results showed that the students in the experimental group of each grade generally performed much better in piano learning than the students in the control group, which proves that the “Pygmalion effect” can play an excellent interventional role in piano teaching. In this research, the value of “Pygmalion effect” in the educational psychology of piano music education in primary schools was deeply studied to explore the possibility of a novel piano teaching model. The research results show that teacher encouragement can stimulate the subjective initiative of students and make them perform better in piano learning. This research provides reference and ideas for the combination of education- and psychology-related research in the music classroom.

## Introduction

Currently, in the process of piano teaching, the classroom format is still based on the traditional “one-to-one” style. In this student-teacher, one-to-one piano teaching classroom model, the teachers play the main role by teaching students their own experience as much as possible to carry out inspiration and demonstration teaching. Students then spend most of their time listening and imitating in a passive learning state, lacking the opportunity to think independently (Xia, 2020). Piano learning largely depends on how the students practice after class to consolidate classroom teaching content. Thus, if students do not have a proactive learning state and the ability to think and learn independently in the exercises after class, the learning process and interest of students in piano learning will be directly affected. Especially for children in the enlightenment stage, such a phenomenon is more prominent. In the music education curriculum of compulsory education in primary and secondary schools in China, piano teaching takes the form of group lessons; that is, the piano teaching classroom model in the form of “one-to-many” (Yang, 2020). However, the “large class” classroom model reflects certain drawbacks. Teachers cannot give every student the same attention within the limited class time. To ensure the effective progress of the music classroom, teachers also tend to focus on students with a positive attitude toward music learning; thus, the feedback for students with negative attitudes toward music class cannot be treated well. For students with such negative learning attitudes, teachers will form lower expectations of their learning ability, and feedback to students through various explicit and implicit behaviors. These students are also pardoned far less than students with a positive learning attitude, reflected by the frequency of them answering questions and intentional or unintentional language from the teacher (Comeau et al., 2019; Xie, 2019). Over time, the self-recognition of students and different treatments from teachers will subtly form self-positioning, which will inevitably lead to learning differences between students (Rickels et al., 2019).

The “Pygmalion effect” suggests that “When a person receives encouragement, praise, and expectations from others, he feels that he has received social support and his self-confidence is enhanced. Then, a positive motivation is formed, he will try to avoid the disappointment of the other party and strive to achieve the expectations of the other party.” “Pygmalion effect” can effectively reduce such differences among students and play a better guiding role in the important initial stage of the formation of the music learning attitudes of primary school students, making the self-positioning of these students develop in a positive direction (Taheri et al., 2019; Espigares-Pinazo et al., 2020). This work tries to apply the “Pygmalion effect” to piano teaching from the perspective of educational psychology, especially for students with a negative attitude toward piano teaching. Its purpose is to enhance the enthusiasm and attitudes of students toward music learning. Moreover, it tries to better optimize the effect of piano teaching based on the mental health development of learners, so that the learning process becomes a virtuous circle.

There are two innovations in the research.

First, psychology research was combined with piano teaching, and a questionnaire was designed for the piano learning of students based on the factors that affect the attitudes of these students toward piano learning.

Second, a piano teaching experiment design based on the “Pygmalion effect” was proposed and applied to the piano teaching class. Then, a control group was set up to verify the actual effect.

Music education is an important part of aesthetic education in the overall development of people, and it plays a very important role in the development of the cognitive, emotional, and behavioral skills of students. Through the psychological research of piano learning, teaching methods suitable for the learning psychology of students were selected to cultivate interest of learners in learning piano. To cultivate all-round, high-quality talents, it is important to strengthen teaching methods, respect individual differences, improve interest of students in piano learning, and promote the teaching effect and quality of music classrooms.

## Current Research Analysis And Literature Review

In the research on piano education, Yang (2021) studied the application of computer audio technology in piano education and explored how to computerize audio materials in piano lessons, so as to maximize the role of music classrooms and mobilize the positive emotions of students. Chen et al. (2021) investigated the status quo of teachers and the cognition students have of information regarding piano education in a certain university. The study also explored how to arm traditional classroom teaching with piano “micro lessons” that use new media to build a networked piano learning environment and a “MOOC” platform for piano teaching. Wang (2021) explored innovative methods and paths suitable for collective piano teaching in colleges and universities based on the background of the era of big data. Li (2020), in the context of the new curriculum reform, combined the training of innovative thinking ability with piano course education and analyzed the training of piano course education and innovative thinking ability. Dai (2019) discussed the innovation path of University piano education teaching reform in his research. Finally, Diana (2019) studied piano teaching scenes in India and the Philippines, trying to find new methods to promote piano teaching by comparing different cultures.

In-depth discussions were made on how to put forward new innovations in the piano collective lesson teaching model in the context of social background, University curriculum education, teachers and students themselves, etc. Then, we explored ways to develop the teaching ability of piano collective lessons, aiming to effectively improve the professional ability of piano education teachers in colleges and universities, thereby improving the level of piano education in China. In summary, the research on piano education mainly focuses on three directions. First, it discusses new piano teaching methods in combination with computer technology. Second, it explores a new classroom format to improve classroom efficiency by mobilizing the positive emotions of students in the classroom. Third, it optimizes the teaching mode of the piano classroom for students in the form of data analysis based on the background of big data.

The purpose of this research is also exploring the new piano classroom model. The value of this research lies in the combination of educational psychology and traditional piano teaching. Based on psychological methods, the enthusiasm of students in the classroom is mobilized, and the piano classroom teaching mode is optimized to achieve better classroom effects.

## Methodologies

### Theoretical Analysis of Educational Psychology

#### Implementation Mechanism of Rosendahl Effect

In many studies on teacher expectation process and theoretical model construction, the research focuses mainly on teachers and how they form and transfer expectations. In the process of theoretical model construction, researchers have proposed different expected action process models (Sha, 2019; Poulter and Cook, 2020). Under the mechanism of “Pygmalion effect,” teachers can obtain all aspects of information on students by directly observing behavior, collecting academic information, and listening to feedback from other teachers and students. This is done by combining past experience to predict the follow-up development of students and form certain expectations based on information sources (Lin and Qun, 2019). The expectations that teachers have of students in different directions can either have a positive or negative impact on students (Marlies et al., 2021).

#### Analysis of Factors Influencing the Attitude of Students Toward Piano Learning

The characteristics of the psychological development of students determine the psychological characteristics they have when engaging in piano music learning. From the perspective of psychological development, children refer to elementary school children in a narrow sense, generally 6 or 7 to 11 or 12 years old, which is called childhood. Erikson's eight-stage theory of personality development reveals that elementary school children are at the stage of acquiring a sense of diligence to overcome inferiority at the age of 7–12. At this stage, it is very important to cultivate self-confidence in students to protect their mental health. In the music curriculum, it is advocated to perceive the elements of music with the body, which requires the participation of children. Throughout the childhood stage, children are also examining and exploring their own abilities. When they find that they are better than others in a certain aspect, or have special interests, they are more willing to participate in activities that they think are suitable for them, so that certain skills can be more fully utilized (McCrudden and Marchand, 2020). In this regard, the attitude of parents and teachers is particularly important. They must be good at providing children with opportunities to make various choices (Fan, 2019).

The factors that affect the piano music learning of students are based on their own physical and psychological development rules. At the same time, there are also external factors that lead to differences in the attitudes students have toward piano music learning (Guven, 2017). The family, as the first environment that children come into contact with, has a subtle influence on their music learning. However, school teaching activities are the main approaches for children to acquire knowledge, and as the main place for children to carry out learning activities, they occupy most of their time. Therefore, the school environment has a decisive influence on the learning of children (Akhavan Tafti et al., 2019; Wallace and Giles, 2019). Thus, this work will focus on two aspects of that affect the attitudes of primary school students toward music learning: family and school factors.

The factors that affect these attitudes toward music learning imply multiple dimensions. The first is the initial impact of family environment factors on children. Then, in the general environment of school music education, the attitude of the school toward music subjects, attitudes of the teachers toward students, other factors that affect teachers, classroom teaching process, etc. all affect the attitudes students have toward music learning (Burak, 2019; Powell, 2019; Aubele, 2020). Therefore, only by analyzing the multi-dimensional factors that affect the music learning of students can the “Pygmalion effect” be truly and effectively applied to primary school music education and change the attitudes of students toward music learning (Emery and Anderman, 2020; Nolen, 2020).

In summary, psychological factors and psychological development have a huge impact on learning interest and learning effect in the process of students learning music. Learning music, in turn, will also play a certain role in promoting and regulating the development of the mental health of students. The combination of music learning and psychology applies the theoretical knowledge of educational psychology to the professional knowledge of learning in the music classroom, making music classroom teaching methods more in line with the physical and mental development laws and psychological characteristics that students are subject to. This research fully mobilizes the enthusiasm students have for learning music, enables students to exert strong learning enthusiasm and subjective initiative, promotes the teaching atmosphere of music classroom, and plays a good classroom effect. As one of many musical instruments, piano should be taught in a way that follows the psychological methods and theories of music teaching. In this study, the application of educational psychology and piano teaching is implemented, the interactive behavior of teachers and students in piano classroom teaching is analyzed by designing questionnaires and setting up control experiments, and the optimization methods of piano classroom teaching are discussed from the perspective of psychology.

### Teaching Experiment Design

#### Design and Reliability and Validity Test of Piano Learning Questionnaire for Students

The questionnaire utilized in this study was based on relevant domestic and foreign research and designed according to the research theme of this article. The questionnaire is divided into five parts with a total of 40 survey questions. The first part is a survey of the attitudes students have toward music learning, including their attitudes toward music, music lessons, and music teachers. The second part is a survey of the psychological level of these students, including self-efficacy, self-concept, and self-esteem level. The third part is a basic understanding of music literacy, including music preferences and participation in the art clubs or extracurricular music training classes in school. In the fourth part, the influence of family environment factors on the attitudes students have toward music learning was also included in the scope of investigation. This included studying the relationship between students and their parents and the possible positive motivational effect parents have on the music learning of their children. A total of six indicators were used in this section.

The scoring method of this questionnaire adopted the Likert scale to test the contents of these four major parts, with the larger the number of the selected result indicating the more consistency with the actual situation. In addition, the reverse questions in the questionnaire were scored in reverse, so that the higher the score, the better the interest and effect of piano learning.

The test results are shown in Table 1.

**Table 1:** Reliability test results.

**Name**	**Total correlation number of correction items (CITC)**	**Term deleted α coefficient**	**Cronbachα coefficient**
Q1	0.713	0.825	0.873
Q2	0.722	0.818	
Q3	0.673	0.863	
Q4	0.711	0.831	
Q5	0.735	0.809	
Q6	0.721	0.822	

*Q1–Q6 represent the six indicators of the questionnaire, respectively*.

#### Experimental Design of Piano Teaching Based on “Pygmalion Effect”

The ultimate goal of the experiment was to reveal that students have positive changes in themselves due to positive expectations from teachers. Through experimental research, the “Pygmalion effect” was adopted to the students in the experimental group, and the students in the control group maintained their original state, proving that the application of “Pygmalion effect” by teachers in the “one-to-many” piano teaching classroom model has a positive impact on the piano learning of students.

The selected experiment site was a central primary school in Beijing City, China. Before the experiment started, three piano music teachers who have served as teachers in three piano teaching classes in their respective grades were selected from the second, fourth, and sixth grades of the school. A total of 124 students in these nine classes were tested on their piano learning problems. According to the measurement results, five students with low scores in each class were selected as the experimental group of each grade. With the setting of 1 experimental group and 2 control groups in each grade, the students were divided into nine groups with a total of 45 students. The experimental group was group A in grade 2, group B in grade 4, and group C in grade 6. The remaining two classes in each grade served as control groups, namely, A1 and A2 groups in grade 2, B1 and B2 groups in grade 4, and C1 and C2 groups in grade 6.

#### Pre-test Questionnaire Survey Procedure

After the consent of the school and related departments was acquired, firstly, the three piano classes in grade 2, 4, and 6 of the school were sampled by the whole class to conduct a questionnaire survey. A total of 146 paper questionnaires were issued with 132 questionnaires returned. Of these questionnaires, eight invalid questionnaires were removed and 124 valid questionnaires were obtained. The effective rate of the questionnaire was 84.9%. There were 124 pieces of data input into the SPSS to test reliability and validity. After the questionnaire reliability and validity were proven qualified, the previous test results were taken as the basis. The questions in the questionnaire were counted in the form of scoring and five students with the lowest scores in each class were sorted out; that is, the students with low interest, self-efficacy, and self-concept in music class. A total of 45 students selected, with five students in experimental group A in grade 2, five students in the control group A1, and five students in the control group A2. In grade 4, there were five students in the experimental group B, five students in the control group B1, and five students in the control group B2. Grade 6 provided five students in experimental group C, 5 in control group C1, and 5 in control group C2.

#### Piano Teaching Experiment Process

The experiment period was one semester. During the 4-month experiment, teachers were guided on how to intervene with the students in the experimental group. The results of the post-test questionnaire were also quantified in the form of integral addition, and numerical values were used as indicators to compare the changes in the attitudes of students toward music. SPSS 25.0 was adopted to perform descriptive statistical analysis on the data obtained from the field survey. Teachers were accompanied the whole process of the questionnaire survey to ensure the authenticity of the questionnaire.

The experimental intervention process started from the personal characteristics of the three teachers A, B, and C corresponding to the three grades; which means that both the external and internal dimensions were considered. The verbal behaviors of teachers such as questions, requests, evaluations, and responses, and non-verbal behaviors such as facial expressions and eye contact were controlled. The students in the control group were not interfered with, and the original state was maintained; if necessary, the students could talk with the teacher for assistance.

#### Test Design of Piano Teaching Classroom Effect

According to experimental observation of the three teachers on the teaching content of the semester, the piano performance evaluation of the students in the experimental group and the control group at the end of the semester was carried out to obtain a comparison of the teaching effect based on the “Pygmalion effect” piano teaching experiment.

The repertoire for the second-year students taught by teacher A was the Czerny 599 etude, number 87, which was arpeggio in F major and presented a very bright and cheerful emotion. At the same time, the expression mark *dolce* also represented that its melody was lyrical and soft. Clean and granular touch keys were played to pop out transparent timbres. At the same time, the changes in the intensity of the music, harmony color, and the difficulties in arpeggio techniques were paid attention to.

The grade 4 students who were taught by teacher B learned the repertoire of Hanon #4. Hanon was a practice item that focused on developing the ability of fingers, which required students to raise their fingers independently, release the keys, relax their arms, and change their fingers in time to really exercise on the finger itself. The slow practice encouraged good finger movement, high finger, small outbreak, independent movement fingers, etc. Every step had to follow strict requirements so that one could finally play smoothly. Hanon #4 was a special practice for 3, 4, and 5 fingers, which are also the three weakest fingers in the human body; thus, having both representative meaning and practice meaning. The reason fast performance was required was that it could intuitively and obviously expose numerous problems such as finger running, sense of rhythm, coordination ability of various parts of the body, and so on.

The repertoire for the grade six students taught by teacher C was the third Bach minuet, which initially met the requirements for a small piece of music. The switching of the music interruption to legato, the repeated occurrence of thematic subtopics, and the interaction of the left-hand and right-hand sentences were the main points of playing, and the technical difficulties were the focus of the investigation. In addition to the technical part, the minuet music was also dynamic, which can examine the sense of rhythm students have in four or three beats, how to deal with melody fluctuations, and how to control the changes in phrases. Theoretically, it was relatively simple to have only two voices after the lesson. The students dividing the musical sections and the sentences in the sections, the theme switching between the left and right hands, and the transformation of the theme itself were the highlights. On the whole, it was a small piece of music that was relatively complete in all aspects and was more suitable for investigation.

The above three experiments had three reasons for choosing different piano music teaching as the research object. First, the music classroom teaching style and areas of expertise of each teacher are different. Therefore, the research process should cover as many aspects as possible to reduce the error caused by the research object. Second, the main purpose of this research is studying the application and effect of educational psychology in piano teaching. To obtain reliable research results, the selected piano music must be representative. The three sets of piano music selected in this study, which were representative of a certain degree, had different technical difficulties and various requirements for learners, who cover three different grades of high school. Third, the selected track needed to have a certain degree of difficulty, so that the research effect can be better realized. To summarize, out of consideration of the above three points, the above three groups of music were selected as the research objects.

### Analysis and Discussion of Questionnaire Survey and Control Experiment Results

#### Design, Reliability, and Validity of Student Piano Learning Questionnaire Test Results

The results of the validity test are shown in Table 2.

**Table 2:** Validity test result.

**KMO**	**0.85**
Bart sphericity	1826.5
Df	113
*P*	0.000

The reliability test of the questionnaire adopted the Cronbach Alpha value, and the validity test adopted the Kaiser–Meyer–Olkin (KMO) value and the Bartlett sphere test value. The Cronbach Alpha value of the student piano learning questionnaire and the Cronbach Alpha value based on standard items were both >0.8, indicating that the inherent reliability of the student piano learning questionnaire was favorable. The KMO value of the questionnaire was >0.8, and the significance of the associated probability of the Bartlett sphericity test was 0.000 <0.001, reaching the significance level, so the validity test of the questionnaire was better.

### Descriptive Statistical Analysis of the Results of the Pre-test Questionnaire

The pre-test questionnaire collected the results of 124 students and their attitudes toward piano learning. In the 124 questionnaires, for the question “I think taking piano lessons is a very happy thing”: 1% of the students chose to disagree completely, 2% of the students chose to disagree relatively, 2% of the students chose to be uncertain, 10% of the students chose to agree more, and 85% of the students chose to agree completely. It can be seen that most students think that taking piano lessons is a very happy thing.

In the 124 questionnaires, for “I was very active in piano lessons”: 19% of the students chose to completely disagree, 11% of the students chose to disagree, 25% of the students chose not to agree, 14% of the students chose to agree, and 31% of the students chose to agree completely. Only 44% of students had a positive attitude toward piano lessons, and more than half of the students believed that their performance in piano lessons was not positive.

### Correlation Analysis of Factors Affecting Piano Learning Attitudes of Students in Pre-test Questionnaire

The results obtained from the pre-experimental test data showed that most students liked piano lessons very much. However, the data also showed that not all students liked to take piano lessons, and more than half of the students were unable to actively participate in the piano classroom learning and interaction. A correlation analysis of the attitude students have toward piano learning was then performed from the two aspects of psychological factors in teachers and in students. The results of the analysis are shown in Table 3. Table 3 shows the relationship between encouragement from teachers and activity of students in learning the piano. There were 124 survey results. According to Table 3, the Pearson correlation coefficient (two-tailed) is 0.427^**^, indicating that the encouragement level of the piano teacher is significantly correlated with the degree of enthusiasm student have in piano lessons at the level of 0.01. Teachers having positive expectations for students made the piano learning attitudes of students tend to be positive, and there was a notable correlation between the two. It was suggested that the research idea of this research is reasonable, and it can influence the enthusiasm of students for learning piano by improving encouragement from teachers. Table 4 shows the relationship between confidence level of students and piano learning activity. In Table 4, the Pearson correlation coefficient (two-tailed) between student confidence level and piano learning activity is 0.276^**^, indicating that there is a significant correlation between self-efficacy and student piano learning enthusiasm at the 0.01 level. It shows that the self-efficacy of students can influence their enthusiasm to learn piano.

**Table 3:** Correlation analysis between encouragement degree of teachers and piano learning activeness.

		**Piano teacher encouragement level**	**Positive degree of piano lessons**
Piano teacher encouragement level	Pearson correlation	1	0.427^**^
	Significance (two-tailed)		0.000
	Number of cases	124	124
Positive degree of piano lessons	Pearson correlation	0.427^**^	1
	Significance (two-tailed)	0.000	
	Number of cases	124	124

** *At 0.01 level (two-tailed), the correlation is significant*.

**Table 4:** Correlation analysis between confidence level of students and piano learning activeness.

		**Student confidence level**	**Positive degree of piano lessons**
Student confidence level	Pearson correlation	1	0.276^**^
	Significance (two-tailed)		0.000
	Number of cases	124	124
Positive degree of piano lessons	Pearson correlation	0.276^**^	1
	Significance (two-tailed)	0.000	
	Number of cases	124	124

** *At 0.01 level (two-tailed), the correlation is significant*.

### Comparison of Piano Learning Attitudes of Middle School Students Before and After the Experiment

**Figure 1:**
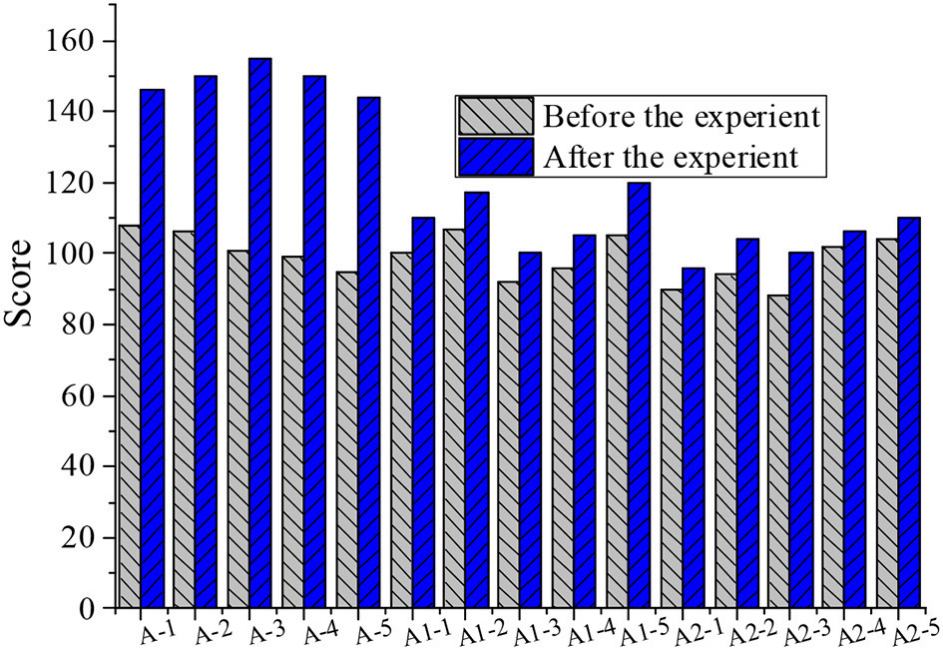
Comparison of piano learning attitudes between groups before and after the experiment in grade 2 (A refers to the experimental group A, A1 refers to the A1 control group, and A2 refers to the A2 control group. −1,−2,−3,−4, and −5 refer to the numbers of the five students, respectively).

**Figure 2:**
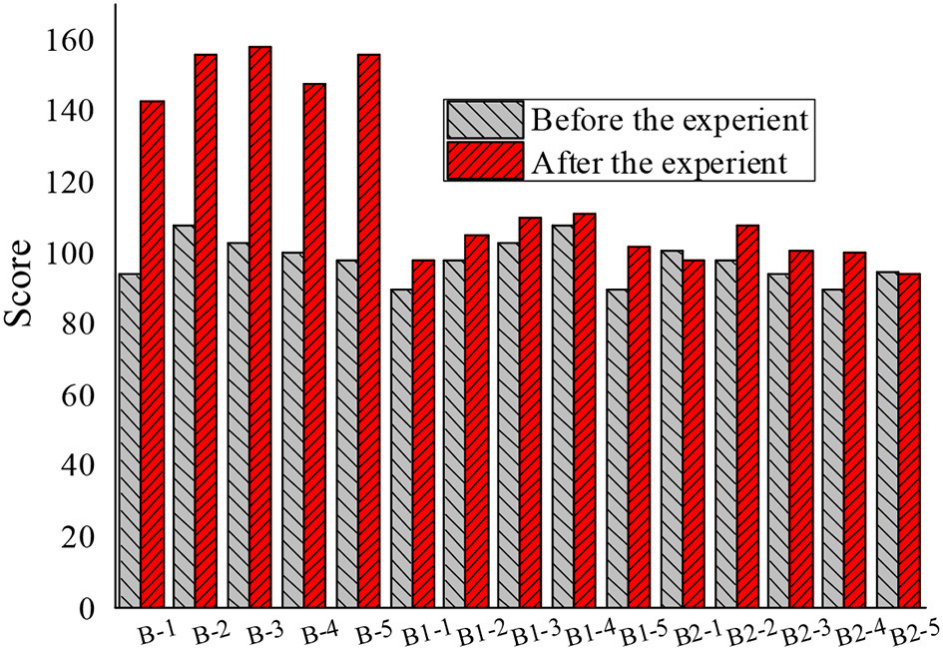
Comparison of piano learning attitudes between groups before and after the experiment in grade 4 (B refers to the experimental group B, B1 refers to the B1 control group, and B2 refers to the B2 control group. −1,−2,−3,−4, and −5 refer to the numbers of the five students, respectively).

**Figure 3:**
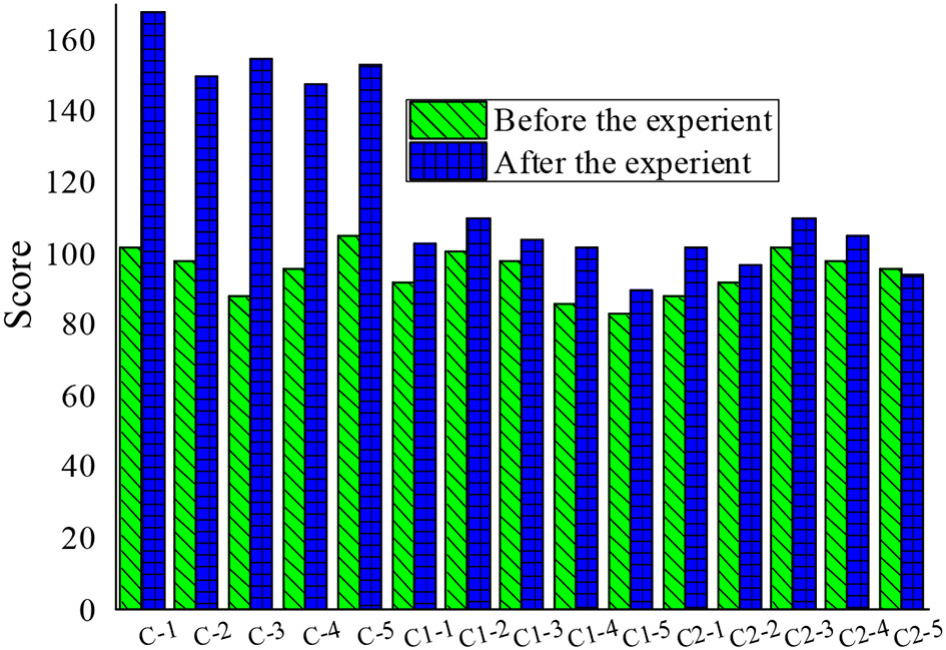
Comparison of piano learning attitudes between groups before and after the experiment in grade 6 (C refers to the C experimental group, C1 refers to the C1 control group, C2 refers to the C2 control group. −1,−2,−3,−4, and −5 refer to the numbers of the five students, respectively).

After the experiment, 45 students in the experimental group and the control group of each grade were re-investigated through the questionnaire. Through the statistics of the piano learning attitude of 45 students in grade 2, 4, and 6, the value of each question in the questionnaire was sequentially added to obtain the data result after quantitative calculation. The total score of the 40 questions in the questionnaire was 160 points, where the higher the score, the more positive the piano learning attitude. Then, the data before and after the experiment were compared, as shown in Figures 1–3.

The 15 students in the experimental group of each grade showed a positive change in their attitude toward piano learning. Due to the individual differences of students, the perception sensitivity of each student to the expectations of the teachers was also different; similarly, since the three grades were taught by different teachers, the ways teachers convey expectations were different. Due to various reasons, teachers were unable to convey all the expectations of each student in the teaching process. Therefore, the degrees of attitude change of different students were also different. Judging from the data results, the results of the questionnaire proved the initial hypothesis of the experiment: the application of “Pygmalion effect” in elementary school music education has a positive meaning for the attitude of students toward piano learning. The results of the piano classroom questionnaire survey between the experimental group and the control group were compared and, after the “Pygmalion effect” intervention, the enthusiasm of learning piano of students in the experimental group A, B, and C significantly improved. The enthusiasm for learning piano of students in the control group, A1, B1, C1, A2, B2, and C2 also increased slightly, but not as obvious as with the “Pygmalion effect” intervention.

### Pygmalion Effect-Based Piano Teaching Experiment

At the end of the experimental observation semester, teacher A tested the learning achievements of Czerny 599 Etude Article 87 of the three piano teaching classes of the second grade. The piano learning results of five students in the experimental group and 10 students in the two control groups were mainly recorded, and the performance effect together with teacher A was evaluated. The comparison results are shown in Table 5. The score of the experimental group was much higher than the scores of students in group 1 and group 2 as the control group. The test scores of the experimental group all reached 4–5 points. However, the control group 1 and the control group 2 did not show obvious advantages in the overall score.

**Table 5:** Comparison of piano teaching effects among groups in grade 2.

**Repertoire points**	**Test group**		**Control group 1**		**Control group 2**	
	**Good**	**Poor**	**Good**	**Poor**	**Good**	**Poor**
Allegretto speed	4	1	2	3	1	4
Stress in sentences	5	0	3	2	4	1
Three times higher octave	5	0	5	0	4	1
Crescendo	4	1	3	2	2	3
Treatment of sentences	4	1	0	5	0	5
Perception of and tone	5	0	1	4	0	5

Teacher B examined the learning results of Hanon IV of three piano teaching students in fourth grade and emphatically recorded the piano learning results of five students in the experimental group and 10 students in the two control groups. The performance effect together with teacher B was evaluated, and the comparison results are shown in Table 6. In the fourth grade, the scores of the experimental group on repertoire performance were much higher than those of the two control groups. Due to the high requirements for finger independence in the performance of the repertoire, the score for this item was only two points, and the remaining scores were three points or more. The lowest finger independence score was still higher than that of groups 2 and 3 as the control group.

**Table 6:** Comparison of piano teaching effects among groups in grade 4.

**Repertoire points**	**Test group**		**Control group 1**		**Control group 2**	
	**Good**	**Poor**	**Good**	**Poor**	**Good**	**Poor**
Smooth playing	4	1	4	1	3	2
Stable rhythm	3	2	3	2	4	1
Wrist relaxation	5	0	2	3	2	3
Finger up burst	4	1	4	1	3	2
Inter-tone granularity	4	1	2	3	2	3
Finger independence	2	3	1	4	1	4

Teacher C tested the learning results of Bach minuet Number 3 of three piano teaching students in grade 4. The piano learning outcomes of five students in the experimental group and 10 students in the two control groups were emphatically recorded, and the performance effects together with teacher C were evaluated. The comparison results of the effects are shown in Table 7.

**Table 7:** Comparison of piano teaching effects among groups in grade 6.

**Repertoire points**	**Test group**		**Control group 1**		**Control group 2**	
	**Good**	**Poor**	**Good**	**Poor**	**Good**	**Poor**
Staccato switching	4	1	2	3	1	4
Crescendo grasp	5	0	3	2	4	1
Difficulties of triplet	5	0	5	0	4	1
The rhythm of dance music	4	1	3	2	2	3
Perception of music	4	1	0	5	0	5
Echoing of hands	5	0	1	4	0	5
Division of voice	5	0	4	1	4	1

The scores of the experimental group in repertoire performance were also higher than those of the control group 1 and the control group 2. Since senior students had stronger comprehension abilities, the overall scores were higher. The experimental group showed relatively better effects, and scores on several items such as perception of music and voice division were much higher than those of the control group. Students in the experimental group of each grade generally performed much better in piano learning than those in the control group. This result fully proves that the “Pygmalion effect” has shown a very good intervention effect in piano teaching.

In summary, the “Pygmalion effect” proposed in this study can be applied to the piano classroom teaching process to improve the classroom participation and enthusiasm of students, and optimize the piano classroom teaching effect. In the survey of teachers in Taiwan on teaching adjustments and learning reflection difficulties of students, researchers found that most analyses of classroom effects by teachers mainly focused on analyzing the methodological errors of students. They often believed that repeated practice by learners was the best way to improve performance (Yeh, 2018). However, this research is from the point of view of the teacher, which combines educational psychology with piano classroom teaching to optimize the teaching.

## Conclusion

During 4 months of experimentation, this work tried to adopt “Pygmalion effect” in piano lessons by using verbal and non-verbal behaviors to convey positive expectations to students to improve their psychology. The results show that encouragement from teachers, self-efficacy of students, and parenting styles were all considerably correlated with the piano learning enthusiasm of students. By comparing the results of the pre-test and post-test of the experiment, the students in the experimental group were notably more motivated to learn piano lessons. The experimental results prove that the adoption of “Pygmalion effect” in piano teaching has remarkable effect on changing the attitudes of students toward piano learning and cultivating self-confidence in these students. Moreover, in the later stage of the experiment, students in the experimental group generally showed excellent piano learning effects. The results show the influence of “Pygmalion effect” on piano learning, and the ingenious application of educational psychology principle can improve the learning effect of piano and other musical instruments.

Piano teaching design is an important way of determining the direction of the piano classroom. It not only reflects the teaching ability of the teacher, but also carries the transmission of verbal and non-verbal behaviors. In teaching design, teachers can give full play to their subjective initiative, analyze the psychology of their students, and make predictions about the possible states of students. Moreover, they can make judgments on these presuppositions in order to actively respond to the state of the student and give timely feedback; thus, conveying positive expectations to students through teaching and helping them develop in a positive direction.

The study of learning motivation has always been a key and difficult point in the field of pedagogy research. The theory is classified as directly related to values or expectations, depending on the content of the study. This study attempted to find a way to improve the teaching effect by exploring the correlation between different factors and classroom teaching, which is a method of studying learning motivation. Similar research will be common in the field of education and teaching in the future, especially in terms of the correct use of psychological methods to intervene in the classroom, which has a very broad application prospect.

Limited by the research level of the author and the objective conditions of the research, the experimental group and the control group in this study had few samples, so the results of the study have errors. In elementary schools, piano teaching is not common, so sample data collection was also difficult, which has a certain impact on this research. This was the shortcoming of this research. The factor of gender is an important factor that cannot be ignored in piano teaching, but due to the limitation of sample size and research time, this study did not cover it. In future research, we will introduce more influencing factors and conduct more comprehensive research based on actual conditions.

## Data Availability Statement

The raw data supporting the conclusions of this article will be made available by the authors, without undue reservation.

## Ethics Statement

The studies involving human participants were reviewed and approved by Minzu University of China Ethics Committee. The patients/participants provided their written informed consent to participate in this study. Written informed consent was obtained from the individual(s) for the publication of any potentially identifiable images or data included in this article.

## Author Contributions

All authors listed have made a substantial, direct and intellectual contribution to the work, and approved it for publication.

## Conflict of Interest

The authors declare that the research was conducted in the absence of any commercial or financial relationships that could be construed as a potential conflict of interest.

## Publisher's Note

All claims expressed in this article are solely those of the authors and do not necessarily represent those of their affiliated organizations, or those of the publisher, the editors and the reviewers. Any product that may be evaluated in this article, or claim that may be made by its manufacturer, is not guaranteed or endorsed by the publisher.

## References

[B1] Akhavan Tafti M. Taheri Ghaletak F. Mohsenpour M. (2019). Evaluating the quality of master degree thesis of educational psychology graduates. J. Res. Plan. Higher Educ. 25, 87–113.

[B2] Aubele J. W. (2020). Educational psychology at the core: adapting a sustainable method for core journal lists. Collect. Manag. 46, 125–141. 10.1080/01462679.2020.1795773

[B3] Burak S. (2019). Self-efficacy of pre-school and primary school pre-service teachers in musical ability and music teaching. Int. J. Music. Educ. 37, 257–271. 10.1177/0255761419833083

[B4] Chen Y. Zheng N. Saravanan V. (2021). AI based research on exploration and innovation of development direction of piano performance teaching in university. J. Intel. Fuzzy Syst. 40, 3681–3687. 10.3233/JIFS-189402

[B5] Comeau G. Lu Y. Swirp M. (2019). On-site and distance piano teaching: an analysis of verbal and physical behaviours in a teacher, student and parent. J. Music Technol. Educ. 12, 49–77. 10.1386/jmte.12.1.49_1

[B6] Dai X. (2019). Research on reform and innovation path of piano education and teaching in colleges and universities. Int. J. Soc. Sci. Educ. Res. 2, 140–144.

[B7] Diana T. (2019). The piano pedagogy scenes in India and the Philippines: An introductory cross-cultural comparison. Int. J. Music Educ. 37, 390–406. 10.1177/0255761419839169

[B8] Emery A. Anderman L. H. (2020). Using interpretive phenomenological analysis to advance theory and research in educational psychology. Educ. Psychol. 55, 220–231. 10.1080/00461520.2020.1787170

[B9] Espigares-Pinazo M. J. Bautista-Vallejo J. M. García-Carmona M. (2020). Evaluations in the moodle-mediated music teaching-learning environment. Technol. Knowl. Learn. 1–15. 10.1007/s10758-020-09468-0

[B10] Fan J. (2019). Research on piano education from the perspective of music eco-environment psychology. Ekoloji 28, 3281–3289.

[B11] Guven E. (2017). Levels of music performance anxiety and test anxiety of Turkish prospective music teachers in piano exams. Int. J. Music Educ. 35, 154–164. 10.1177/0255761415620530

[B12] Li J. (2020). Analysis of piano curriculum education and cultivation of creative thinking ability. Reg. Educ. Res. Rev. 2, 6–6. 10.32629/rerr.v2i1.85

[B13] Lin C. Qun L. (2019). Thinking and practice of piano teaching mode expansion in colleges and universities. Front. Neuroinform 2, 27–32. 10.25236/FER.020606

[B14] Marlies V. Johnson S. K. Leroy H. (2021). Exploring the bounds of pygmalion effects: congruence of implicit followership theories drives and binds leader performance expectations and follower work engagement. J. Leadersh. Organ. Stud. 28, 137–153. 10.1177/1548051820980428

[B15] McCrudden M. T. Marchand G. (2020). Multilevel mixed methods research and educational psychology. Educ. Psychol. 55, 197–207. 10.1080/00461520.2020.1793156

[B16] Nolen S. B. (2020). Challenging research norms in educational psychology. Educ. Psychol. 55, 267–272. 10.1080/00461520.2020.1810043

[B17] Poulter V. Cook T. (2020). Teaching music in the early years in schools in challenging circumstances: developing student teacher competence and confidence through cycles of enactment. Syst. Pract. Action Res. 1–17. 10.1080/09650792.2020.1765185

[B18] Powell S. R. (2019). Structure and agency in novice music teaching. Res. Stud. Music Educ. 41, 206–218. 10.1177/1321103X18794514

[B19] Rickels D. A. Hoffman I. I. I. E. C. Fredrickson W. E. (2019). A comparative analysis of influences on choosing a music teaching occupation. J. Res. Music. Educ. 67, 286–303. 10.1177/0022429419849937

[B20] Sha L. (2019). Practical research on integration of information technology and kindergarten music teaching. J. Phys. Conf. Ser. 1345:042028. 10.1088/1742-6596/1345/4/042028

[B21] Taheri A. Meghdari A. Alemi M. Pouretemad H. R. (2019). Teaching music to children with autism: a social robotics challenge. Sci. Iran. 26, 40–58. 10.24200/sci.2017.4608

[B22] Wallace F. Giles P. (2019). Participatory research approaches in educational psychology training and practice. Educ. Psychol. Res. Pract. 5, 1–9.

[B23] Wang J. (2021). Innovative research on the teaching mode of piano group lessons under the background of big data. J. Phys. Conf. Ser. 1744:032031. 10.1088/1742-6596/1744/3/032031

[B24] Xia Y. (2020). Resource scheduling for piano teaching system of internet of things based on mobile edge computing. Comput. Commun. 158, 73–84. 10.1016/j.comcom.2020.04.056

[B25] Xie H. (2019). Internet thinking in college music education professional piano teaching. Front. Neuroinform 2,1–4. 10.25236/FER.2019.021201

[B26] Yang H. (2021). Research on the application of computer audio technology in piano education. J. Phys. Conf. Ser. 1915:032074. 10.1088/1742-6596/1915/3/032074

[B27] Yang Z. Y. (2020). Modern piano teaching technologies. Elementary Educ. Online 19, 1812–1819. 10.17051/ilkonline.2020.735171

[B28] Yeh Y. (2018). An investigation of Taiwanese piano teachers' reflection on teaching challenges and pupils' learning difficulties. Music Educ. Res. 20, 32–43. 10.1080/14613808.2016.1249359

